# Identification and characterization of critical values in therapeutic drug monitoring: a retrospective analysis

**DOI:** 10.1038/s41598-024-62402-7

**Published:** 2024-05-21

**Authors:** Yufei Xiao, Lingcheng Xu, Yun Qian, Yang Xu

**Affiliations:** 1 Department of Clinical Laboratory, The Second Affiliated Hospital, Zhejiang University School of Medicine, Hangzhou, China; 2 Department of Pharmacy, The Second Affiliated Hospital, Zhejiang University School of Medicine, Hangzhou, China; 3 Department of Clinical Laboratory, Stomatology Hospital, School of Stomatology, Zhejiang University School of Medicine, Zhejiang Provincial Clinical Research Center for Oral Diseases, Key Laboratory of Oral Biomedical Research of Zhejiang Province, Cancer Center of Zhejiang University, Hangzhou, China; 4 Department of Hematology, The Second Affiliated Hospital, Zhejiang University School of Medicine, Hangzhou, 310009 China

**Keywords:** Therapeutic drug monitoring, Drug concentration, Critical value, Vancomycin, Digoxin, Tacrolimus, Drug safety, Drug regulation

## Abstract

Therapeutic drug monitoring (TDM) is a crucial clinical practice that improves pharmacological effectiveness and prevent severe drug-related adverse events. Timely reporting and intervention of critical values during TDM are essential for patient safety. In this study, we retrospectively analyzed the laboratory data to provide an overview of the incidence, distribution pattern and biochemical correlates of critical values during TDM. A total of 19,110 samples were tested for nine drug concentrations between January 1, 2019, and December 31, 2020. Of these, 241 critical values were identified in 165 patients. The most common critical values were vancomycin trough (63.4%), followed by tacrolimus trough (16.9%) and digoxin (15.2%). The primary sources of drug critical values were the department of general intensive care unit (ICU), cardiology, and surgery ICU. At baseline or the time of critical value, significant differences were found between the vancomycin, digoxin, and tacrolimus groups in terms of blood urea nitrogen (BUN), creatinine, N-terminal Pro-B-Type Natriuretic Peptide (NT-proBNP), and lymphocyte percentage, *P* < 0.05. Therefore, it is important to prioritize and closely monitor drug concentrations to reduce laboratory critical values during TDM.

## Introduction

For many drugs with narrow therapeutic windows or high interindividual pharmacokinetic (PK) and pharmacodynamic (PD) variability, dose adjustment based on therapeutic drug monitoring (TDM) is now a common clinical practice to maintain balance between drug efficacy and toxicity. Currently, TDM involves several categories of drugs, including antibiotics, cardioactive drugs, immunosuppressive agents, antiepileptics, and antipsychotics^1,2^. Vancomycin is a glycopeptide antimicrobial agent used against Gram-positive bacteria, particularly methicillin-resistant Staphylococcus aureus^3,4^. Digoxin has been utilized to treat acute and chronic heart failure and atrial fibrillation for centuries^5^. Tacrolimus, also known as FK506, is a potent immunosuppressant derived from the macrolactam natural product. Tacrolimus binds to FK506-binding protein and inhibits calcineurin, a calcium-dependent serine/threonine protein phosphatase, resulting in suppression of T cell proliferation and activation^6^. When a drug concentration reaches a critical value, a defined threshold that is potentially life-threatening, emergency patient evaluation and intervention are required. Thus, proper management of critical values during TDM is essential to avoid serious drug-related adverse events^7^. Laboratory personnel should promptly report critical values to those directly responsible for patient care, facilitating timely communication between the laboratory and the clinic^8^. Notification and processing of critical values has been reported for clinical chemistry, hematology, coagulation and microbiology^8,9^, but little is known about the management of TDM critical values. Moreover, most TDM studies were restricted to a specific drug category, and there is few data regarding the systemic characterization of critical values across different classes of drugs. In this study, we conducted a retrospective analysis of TDM critical value data to characterize their distribution pattern and clinical correlates. Our focus was on three drugs: vancomycin, digoxin, and tacrolimus.

## Patients and methods

### Patients

The TDM critical values and clinical data of patients were collected from the Second Affiliated Hospital, Zhejiang University School of Medicine. The inclusion criteria were as follows: (1) the assessed drug for TDM had been used for ≥ 3 days; (2) ≥ 1 critical value was reported; (3) the steady-state concentration was obtained; (4) complete clinic data including blood counts and chemistries were available. The exclusion criteria were as follows: (1) lack of a steady-state level; (2) insufficient clinic data. This study followed the ethical guidelines of the Declaration of Helsinki and was approved by Ethics Committee of the Second Affiliated Hospital of Zhejiang University School of Medicine (No. 2021-641). All research was performed in accordance with relevant guidelines and have been performed in accordance with the Declaration of Helsinki. All data generated or analysed during this study are included in this published article.

### Drug concentration assays

Concentrations of vancomycin, digoxin, tacrolimus, cyclosporine, rapamycin, carbamazepine, phenobarbital, phenytoin and valproic acid are detected by immunoassays. Once the drug level reaches a steady-state, blood samples for trough concentration were collected 0–1 h before drug administration. Specifically, serum vancomycin trough concentration was measured by latex agglutination turbidimetry; digoxin concentration in plasma was analyzed by the MULTIGENT Digoxin assay, a homogeneous particle enhanced turbidimetric inhibition immunoassay (PETINIA); tacrolimus concentration in whole blood was measured by chemiluminescent microparticle immunoassay (CMIA).

### Critical values

The items and normal ranges of blood drug concentration and their critical values were adopted based on the survey report of the College of American Pathologists (CAP), combined with the patient safety requirements of the Chinese Hospital Association.

### Statistical analysis

The data were obtained from the Laboratory Information System (LIS) for laboratory blood concentration criticality items, including specimen number, medical record number, requesting department, clinical diagnosis, test items and results, and reporting time. Descriptive statistics were applied to summarize patient and disease characteristics. One-way ANOVA analysis was used to compare clinical parameters between drug groups, while Student's t-test was used to compare data before and after critical values. All statistical analyses were performed with GraphPad Prism software (San Diego, CA). All reported P values were 2-tailed, and statistical significance was set at *P* < 0.05.

### Ethical approval

The studies involving human participants were reviewed and approved by Ethics Committee of the Second Affiliated Hospital of Zhejiang University School of Medicine (No. 2021-641). The patients/participants provided their written informed consent to participate in this study. Written informed consent was obtained from the individual(s) for the publication of any potentially identifiable images or data included in this article.

## Results

### Overview of critical values of blood drug concentration

The reference ranges and critical values for drug concentrations are listed in Table 1. A total of 165 patients were reported to have critical drug levels between January 1, 2019 and December 31, 2020. These included vancomycin (n = 99), digoxin (n = 25), tacrolimus (n = 35), rapamycin (n = 2), phenytoin (n = 2), cyclosporin A (n = 1) and phenobarbital (n = 1). A total of 19,110 blood drug concentration tests were performed, and critical values were reported in 1.3% (241/19,110) of cases. As shown in Table 1, 96.3% (232/241) of the critical values were observed in the following drugs: vancomycin trough 63.37% (154/243), tacrolimus trough 16.9% (41/243) and digoxin 15.2% (37/243). The incidence of critical value was 19.7% for vancomycin, 5% for digoxin and 0.4% for tacrolimus. In contrast, only a small proportion (4%) of critical values were observed for antiepileptic drugs such as carbamazepine, phenobarbital, phenytoin, and valproic acid (Table 1).

**Table 1:** Proportion and incidence of critical values of blood drug concentrations.

Drug concentration item	Reference range (adult)	Reference range (child)	Critical value	CV and its proportion n (%)	Number of tests	CV incidence (%)
Vancomycin (trough) (μg/mL)	10–20	5–15	> 20	154 (63.4)	781	19.7
Digoxin (ng/mL)	0.5–2.0	0.8–2.0	> 2.5	37 (15.2)	738	5.0
Tacrolimus (ng/mL)	5–15	5–10	> 25	41(16.9)	10,791	0.4
Cyclosporine (trough) (ng/mL)	150–600	100–200	> 750	2 (0.8)	1871	0.1
Rapamycin (ng/mL)	5–15	5–15	> 20	3 (1.2)	174	1.7
Carbamazepine (μg/mL)	4–12	4–12	> 20	0	726	0
Phenobarbital (μg/mL)	15 ~ 40	15–40	> 60	1 (0.4)	172	0.6
Phenytoin (μg/mL)	10–20	7–20	> 40	3 (1.2)	150	2
Valproic acid (μg/mL)	50–100	50–100	> 200	0	3707	0
Total				241 (100)	19,110	1.3

### Characterization of critical values in drug concentration

To identify clinical features related to critical values, we summarized the baseline patient characteristics of three major drug groups in Table 2. The vancomycin group comprised 99 patients, the digoxin group 25 patients, and the tacrolimus group 35 patients. The median ages differed between the groups. For instance, patients receiving digoxin treatment were much older than those receiving tacrolimus (71 vs. 41), indicating different disease populations. Digoxin is typically used in elderly patients with heart disease, while tacrolimus is indicated for younger organ transplant recipients. Of the critical values, 83.7% were derived from inpatients and 16.3% were from outpatients. Among the testing items, 28.5% (43/241) occurred more than once, including 30 cases in the vancomycin group. It is noteworthy that the critical values were corrected in 62 of 99 cases of vancomycin, 21 of 25 cases of digoxin, and 33 of 35 cases of tacrolimus groups (Table 2). The median time for corrections was variable, with 24 h (5–331) for the vancomycin, 42 h (23–360) for the digoxin, and 24 h (21–336) for the tacrolimus group.

**Table 2:** Baseline patient characteristics.

	Vancomycin (n = 99)	Digoxin (n = 25)	Tacrolimus (n = 35)
Age, median (range)	58 (2–84)	77 (37–100)	41 (1–72)
Sex (male/female)	59/40	12/13	23/12
Outpatient/inpatient	1/98	8/17	17/18
Disease, n (%)			
	Valvular heart disease, 27 (27.3%)	Heart failure,7 (28%)	Kidney transplant,12 (34.3%)
	Stroke, 7 (7.1)	Valvular heart disease, 3 (12%)	Lung transplant, 6 (17.1%)
	Multiple organ failure, 6(6.1%)	Cardiomyopathy, 3(12%)	Liver transplant, 4 (11.4)
	Renal failure 5 (5.1%)	Atrial fibrillation 2, (8%)	Nephrotic syndrome, 3 (8.6%)
	Pneumonia, 5 (5.1%)	Drug intoxication, 2 (8%	Cardiomyopathy 2 (5.7%)
	Other, 49 (49.5%)	Other, 8 (32%)	Other, 8 (22.9%)
CV episodes (n)	154	37	41
= 1	124	30	35
≥ 2	30	7	6
CV correction			
Yes	62	21	33
No or unknown	37	4	2
Correction time (h)	24 (5–331)	42 (23–360)	24 (21–336)

**Figure 1:**
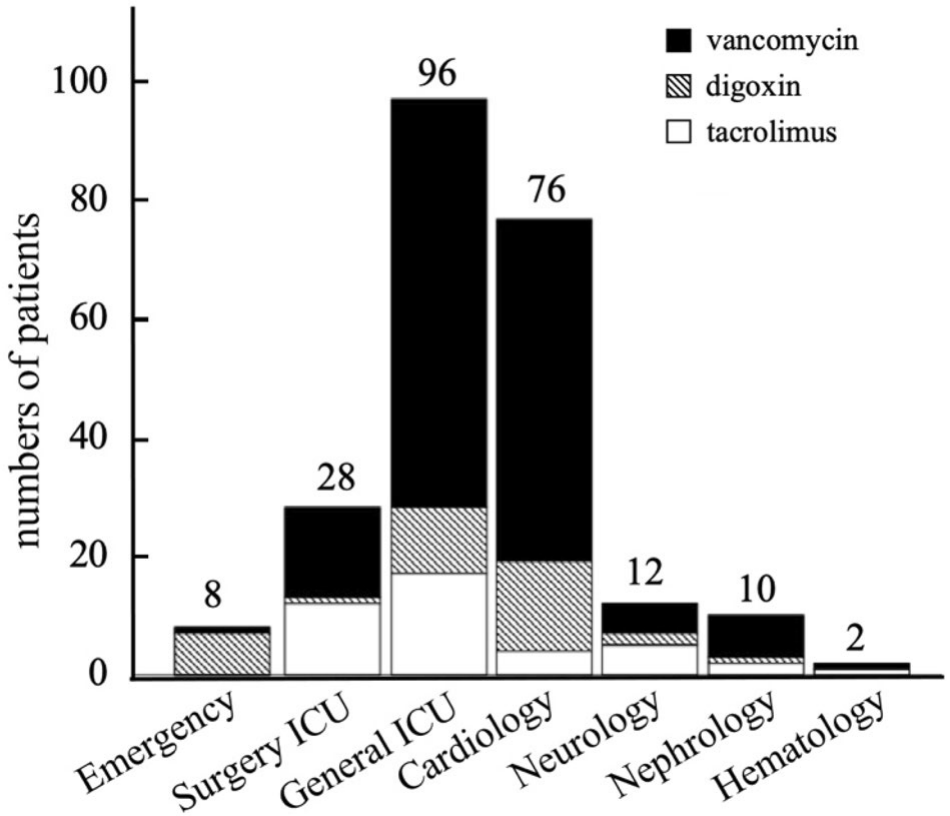
Distribution of critical values in blood drug concentration across the departments in the hospital.

The distribution of critical values across departments is shown in Fig. 1. The majority of patients were from the general intensive care unit (ICU), accounting for 39.9% (96/243), followed by cardiology at 31.3% (76/243) and surgery ICU at 12.8% (28/243).

### Turnaround and reporting times for critical values

**Figure 2:**
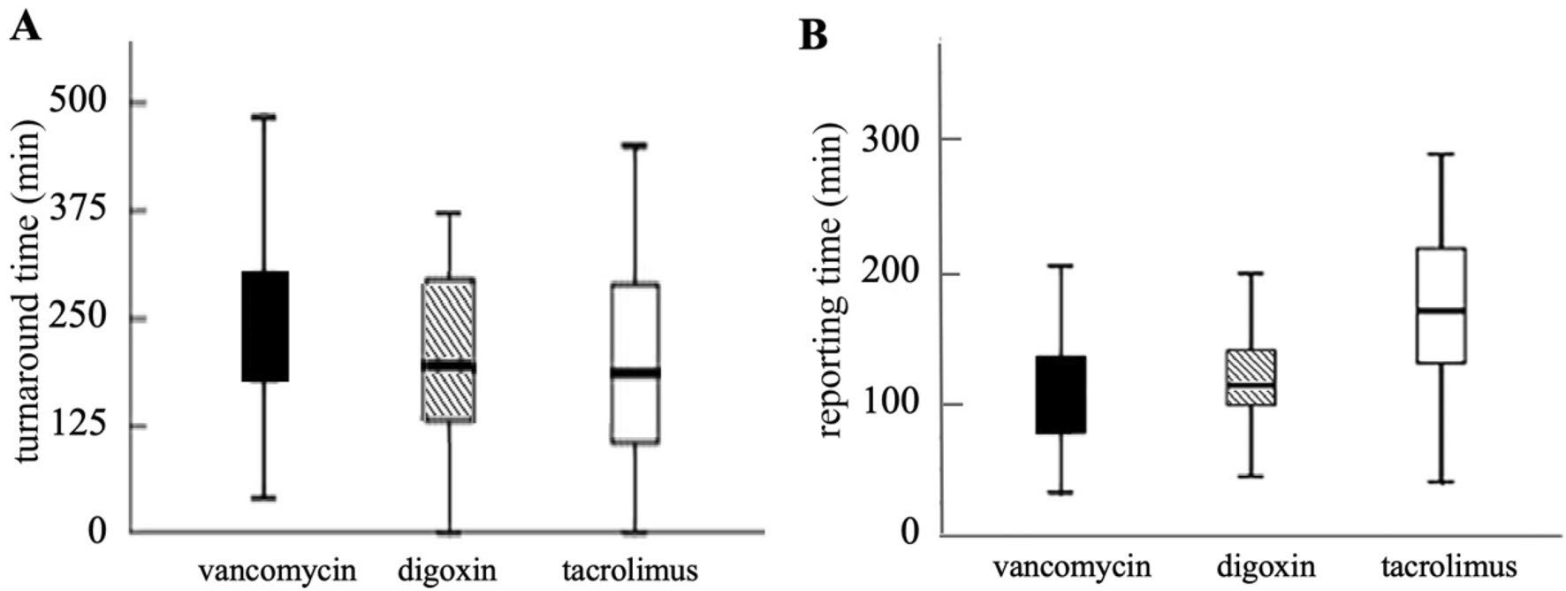
Specimen turnaround time (**A**) and reporting time (**B**) for critical values of vancomycin (trough), digoxin and tacrolimus (trough).

The turnaround and reporting times for each critical value group are summarized in Table 3. The laboratory turnaround time from blood drawing to lab results reporting varied, with 91.4% of critical values having a turnaround time exceeding one hour. The vancomycin (trough) group had a significantly longer turnaround time than the digoxin and tacrolimus groups, *P* < 0.05 (Fig. 2A). The reporting times, defined as the time between sample arrival at the laboratory and results reporting, were 170 and 108 min for FK-506 and vancomycin, respectively. The majority of critical values (89.71%) were reported within 1–3 h (Table 3). The reporting time for tacrolimus was longer than for the other drug groups, *P* < 0.05 (Fig. 2B).

**Table 3:** Turnaround and reporting times for critical values of drug concentrations.

Critical value items	Turnaround (min), median (range)	Reporting (min), median (range)
Vancomycin (trough)	251 (179–302.3)	108.0 (78–137)
Digoxin	195 (108–299)	114 (99–142)
Tacrolimus (trough)	186 (100–292)	170 (127–223)
Others	168 (120–304)	140 (90–250)

### Clinical factors correlated with critical value

**Figure 3:**
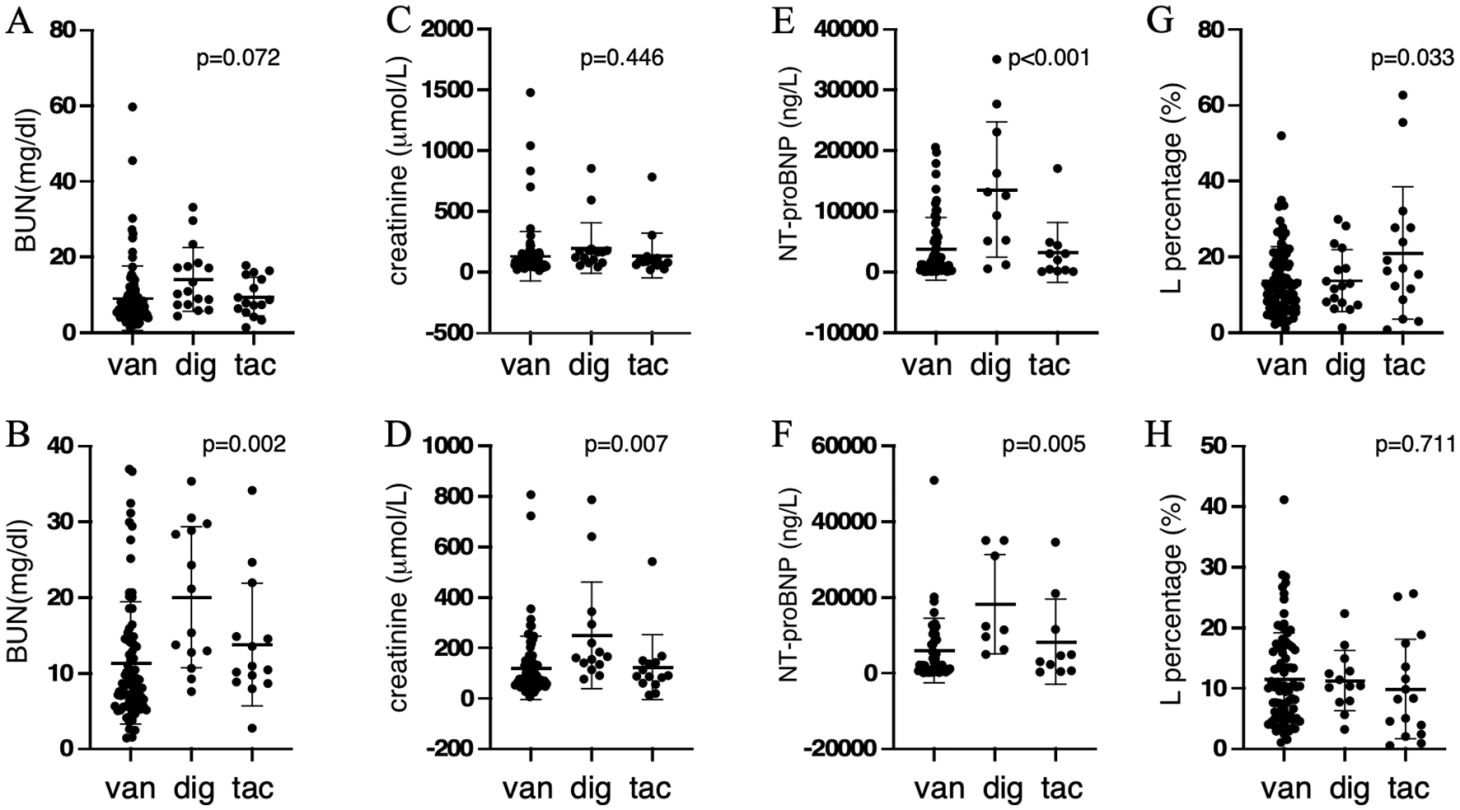
The BUN, creatinine, NT-proBNP and lymphocyte percentage between vancomycin (trough), digoxin; tacrolimus (trough) groups at baseline and at the time of critical value.

**Figure 4:**
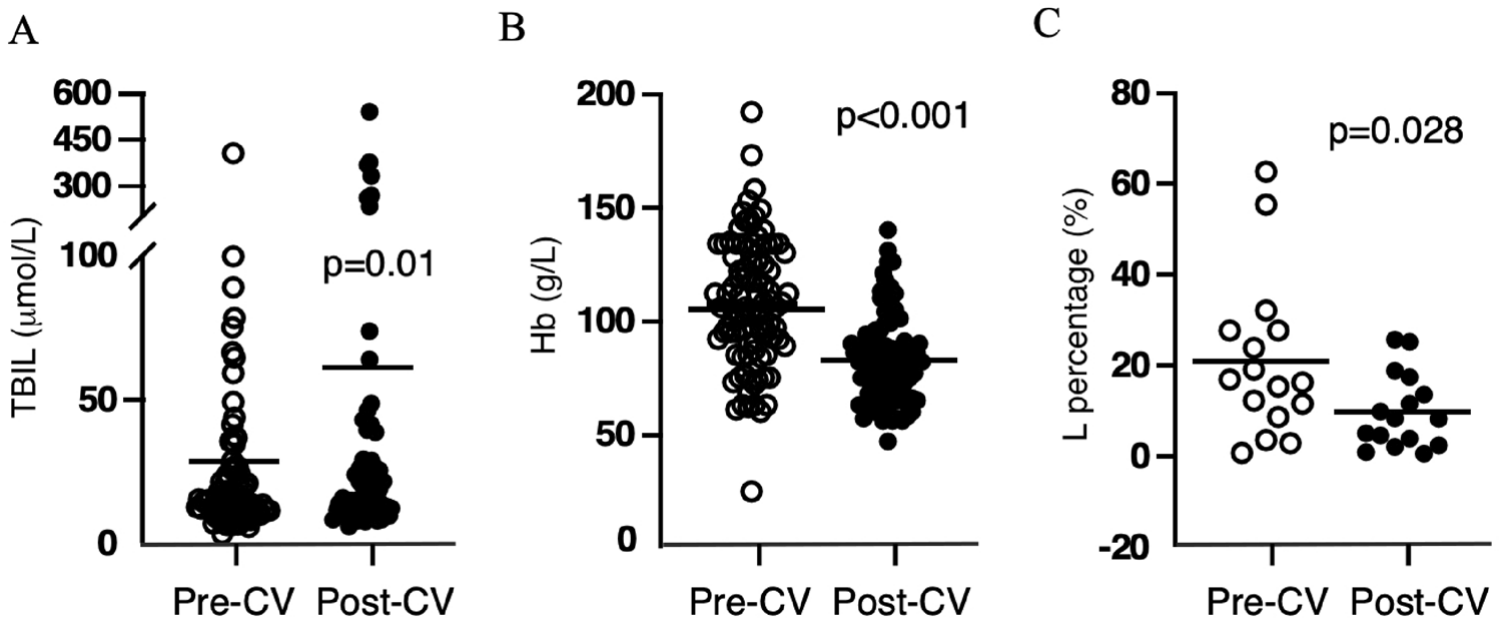
The total bilirubin (**A**) and hemoglobin (**B**) before and after critical value in vancomycin group. The lymphocyte percentage (**C**) before and after critical value in tacrolimus group. CV, critical value.

To determine the clinical factors associated with critical values of vancomycin, digoxin, and tacrolimus, we summarized the results of complete blood count (CBC) and biochemical assays at baseline or at the onset of critical values in Table 4. Four parameters showed significant differences between the three drug groups. At baseline or critical value, the blood urea nitrogen (BUN) level was significantly higher in the digoxin group compared to the vancomycin or tacrolimus group (*P* < 0.01) (Fig. 3A and B). Although the baseline creatinine levels were not significantly different (Fig. 3C), the creatinine level at the time of critical value was significantly higher in the digoxin group than in the vancomycin and tacrolimus groups, *P* < 0.05 (Fig. 3D). As anticipated, the N-terminal Pro-B-Type Natriuretic Peptide (NT-proBNP), a well-known biomarker of heart failure, was significantly elevated in the digoxin group compared to the vancomycin and tacrolimus groups (Fig. 3E and F). The baseline percentage of lymphocytes was higher in the tacrolimus group than in the vancomycin and digoxin groups (Fig. 3G). However, it decreased to a level comparable to the other two groups (Fig. 3H), suggesting that tacrolimus acts as an immunosuppressive agent capable of inhibiting lymphocyte proliferation. Clinical parameters were also compared before and after the development of critical value within each group. There was a significant increase in total bilirubin but a decrease in hemoglobin in patients receiving vancomycin (Fig. 4A and B). In the tacrolimus group, there was a significant decline in the lymphocyte fraction following the critical value, which is consistent with the pharmacological activity of the drug (Fig. 4C).

**Table 4:** Comparisons of laboratory parameters between drug groups at baseline or at the time of critical values.

	Vancomycin (n = 99)	Digoxin (n = 25)	Tacrolimus (n = 35)	*P* value #
WBC (× 109/L)				
Baseline	9.600 (0.7–44.3)	7.2 (4.2–35.2)	6.5 (1.3–21.8)	0.289
Critical value	8.600 (1.2–38.6)	11.1 (5.6–22.1)	8.0 (0.7–19.4)	0.818
N (%)				
Baseline	80.7 (24–97.2)	79.5 (62–92.4)	70.2 (28.9–98.4)	0.082
Critical value	81.9 (42.6–95.7)	82.5 (65.7–91.8)	88.6 (43.5–96.9)	0.371
L (%)				
Baseline	12.50 (1.1–51.9)	12.2 (1.2–29.8)	16.6 (0.7–62.6)	0.033*
Critical value	10.10 (1–41.1)	10.4 (3.2–22.3)	8.25 (0.5–2.6)	0.711
Hb (g/L)				
Baseline	106.0 (25–192)	106.0 (71–204)	98.0 (45–147)	0.371
Critical value	82.0 (47–140)	79.0 (55–174)	84.0 (42–135)	0.207
PLT (× 10^12^/L)				
Baseline	174.0 (23–614)	170.0 (73–898)	211.0 (18–526)	0.579
Critical value	166.0 (2–565)	140.0 (47–1120)	126.0 (12–508)	0.455
TB (mg/L)				
Baseline	14.6 (3.3–405.8)	17.4 (8.7–91.8)	13.1 (4.1–373.4)	0.576
Critical value	18.6 (11.3–540.6)	31.3 (9.4–276.8)	15.5 (4.2–368.2)	0.894
TP (g/dL)				
Baseline	63.15 (26.3–88.6)	63.30 (48.2–43.7)	61.1 (40.3–79.9)	0.932
Critical value	59.8 (28.9–79.1)	60.9 (47.6–73.5)	62.2 (30.3–70.1)	0.529
ALB (g/dL)				
Baseline	32.50 (14–49.7)	32.70 (24.2–42.9)	33.35 (22.0–42.8)	0.795
Critical value	32.95 (16.4–47.2)	33.00 (28.1–38.8)	35.2 (18.6–44.7)	0.742
ALT (IU/L)				
Baseline	29.00 (3–2230)	22.00 (3–106)	31.00 (9–1497)	0.614
Critical value	34.0 (1–1982)	26.5 (8–209)	32.0 (9–595)	0.609
AST (IU/L)				
Baseline	29.00 (5–4480)	37.00 (8–474)	36.00 (13–1802)	0.844
Critical value	41.0 (9–2280)	43.0 (19–305)	38.0 (13–787)	0.748
BUN				
Baseline	6.820 (1.3–59.6)	10.82 (4.3–33.1)	8.2 (1.3–17.7)	0.072
Critical value	8.54 (1.4–36.9)	18.22 (7.6–35.3)	10.65 (2.7–34.1)	0.002
Cr				
Baseline	75.00 (10–1476)	161.0 (38–850)	84.5 (15–780)	0.446
Critical value	81.5 (5–805)	161.5 (75–785)	87.5 (12–540)	0.007
CRP				
Baseline	48.76 (0.7–314.6)	14.6 (4–252.1)	11.5 (1.6–208.)	0.144
Critical value	55.65 (4.4–293.3)	47.0 (5.5–126)	49.50 (5.3–34.9)	0.256
PCT				
Baseline	0.5 (0–88.2)	0.4 (0.1–58.0)	0.2 (0–78.4)	0.993
Critical value	1.450 (0.1–93.1)	2.97 (0.2–19.4)	1.170 (0.1–75.2)	0.856
NTproBNP				
Baseline	1477 (32–20,518)	12,577 (466–35,000)	1923 (15–17,022)	< 0.001*
Critical value	2104 (27–50,852)	11,824 (4927–35,000)	3780 (206–34,475)	0.005*

WBC, white blood cell; Hb, hemoglobin; PLT, platelet; N, neutrophil; L, lymphocyte; TB, total bilirubin; TP, total protein; ALB, albumin; ALT, alanine aminotransferase; AST, aspartate aminotransferase; BUN, blood urea nitrogen; Cr, creatinine; CRP, C-reactive protein; PCT, procalcitonin; NTproBNP, N-terminal pro B-type Natriuretic Peptide. ^#^*P* values were derived from comparison of multiple drug groups using one-way ANOVA analysis. *P < 0.05 considered statistically significant.

## Discussion

In general, candidate drugs for TDM share several pharmacological properties, which include a clear correlation between drug concentration and clinical efficacy, a narrow therapeutic window, high interindividual PK/PD variability, a long duration of therapy, severe overdose consequences and the lack of functioning as active metabolites^1,10^. To ensure patient safety, it is crucial to maintain high-quality testing and analysis throughout the entire process, particularly with regard to critical drug levels, because overdosing can often result in acute toxicities, which can be potentially life-threatening for critically-ill patients^11,12^.

The incidences of laboratory critical values varied considerably among different testing items. Hu et al. identified 601 (0.83%) critical values in electrolytes and glucose from 72,259 routine clinical chemistry specimens^13^. Li et al. expanded the scope of their analysis to include hematology, chemistry, coagulation and microbiology, and reported a critical value incidence of 0.49% (38,020/7,706,962) across all items. Of these, 63% were from inpatients, followed by 24% from emergency department and 13% from outpatient department^8^. There were few data on critical values during TDM. In this study, 1.3% of drug concentration items were reported as TDM critical values, with a higher prevalence in general ICU and cardiology departments. Inpatients accounted for 83.7% of critical values. Notably, critical values occurred more than once in 28.5% of patients, which may be due to ineffective control of underlying causes, highlighting the importance of timely reporting and early intervention.

A recent survey revealed that the most common drugs for TDM in Chinese hospitals include vancomycin, valproic acid, carbamazepine, phenytoin sodium, and methotrexate^14^. Similarly, this study focuses on the characteristics of the three most common critical values during TDM in our hospital. The role of drug trough concentration remains controversial, as the area under the curve (AUC) is a reliable surrogate of cumulative drug exposure, which correlates better with clinical efficacy and toxicity. The TDM guidelines by American and Japanese societies recommend AUC only, whereas the Chinese and European guidelines recommend both AUC and trough concentration for patients receiving vancomycin^15^. Nevertheless, the calculation of AUC is often not feasible in source-limited settings; among critically ill adults without dialysis, vancomycin trough concentrations were associated with AUC^16^. Trough concentrations are still used for routine TDM of tacrolimus in most transplant centers, and the clinical benefits of AUC monitoring over trough level-guided strategies should be evaluated in prospective studies conducted in transplant recipients^6^. TDM of digoxin is highly recommended because of its very narrow therapeutic window. Digoxin toxicity is featured by a heterogeneous and nonspecific constellation of symptoms, including fatigue, confusion, abdominal pain, visual changes, mental alteration, cardiac arrhythmia and worsening of heart failure, which is associated with increased mobility and mortality^17^. Our findings indicated that NT-proBNP levels were higher in the digoxin group, reflecting poor cardiac function observed in patients requiring digoxin treatment.

Laboratory turnaround time is an important parameter for the critical value reporting system. In a closed‐loop non-TDM critical value notification system, the median turnaround and reporting time were 393 min and 41 min, respectively^8^. We found that the majority of critical values in TDM have a turnaround time of 186–251 min and a reporting time of 108–170 min. This is primarily attributable to the complexities of multi-campus management and delayed specimen delivery to the central laboratory, which is located in a single campus within the hospital. To reduce turnaround time, it is essential to streamline sample logistics and develop on-site TDM facilities that integrate novel technologies^12,18,19^.

Currently, the common TDM methods include chromatography, immunoassay, biosensor, electrochemical methods, capillary electrophoresis, and microbial assays^10,20^. In our hospital, we measure all the TDM drug levels with various immunoassays, which are based on the binding of specific antibodies to the drug of interest. Immunoassay is a simple, rapid, cost-effective and high-throughput approach with high specificity and sensitivity, especially suitable for immunosuppressants^2^. Although liquid chromatography (LC), or LC in combination with mass spectrometry (LC–MS), may be more specific and sensitive and take a shorter turnaround time in some cases, they are often limited by high costs, low throughput, facility requirements, and technician training^20^. In addition, the emerging biosensor-based TDM can provide on-site analysis to overcome traditional limitations such as high costs and long turnaround time.

Our study has a few limitations. This is a retrospective analysis with heterogeneous patient cohorts, and the small number of patients in the digoxin and tacrolimus groups may contribute to selection bias and insufficient statistical power, and prospective studies with more patients are warranted. Drug concentrations are clearly influenced by drug-drug interactions and gene polymorphisms of drug-metabolizing enzymes, and further information on concomitant drug use and pharmacogenomic data will provide more insight into the evolution of critical drug levels. Finally, we were not able to fully assess the clinical efficacy of the drugs and did not report the impact of the critical level on patient survival.

In summary, TDM has been a crucial practice that enables physicians to individualize drug treatment while maintaining a reasonable balance between efficacy and toxicity. However, the occurrence of critical values is not straightforward and remains one of the major challenges in clinical practice. We have demonstrated that TDM is susceptible to laboratory critical values. Therefore, it is essential to improve the testing process and enhance reporting and communication of critical values between the laboratory and clinic to ensure patient safety.

## Data Availability

All research data was provided within the manuscript.
